# Predictors of mobility disability in older adults with physical frailty and sarcopenia

**DOI:** 10.1093/geroni/igaf122.3998

**Published:** 2025-12-31

**Authors:** Riccardo Calvani, Matteo Tosato, Stefano Cacciatore, Emanuele Marzetti, Francesco Landi

**Affiliations:** Università Cattolica del Sacro Cuore, Rome, Lazio, Italy; Università Cattolica del Sacro Cuore, Rome, Lazio, Italy; Università Cattolica del Sacro Cuore, Rom, Lazio, Italy; Università Cattolica del Sacro Cuore, Rome, Lazio, Italy; Università Cattolica del Sacro Cuore, Rome, Lazio, Italy

## Abstract

Older adults with physical frailty and sarcopenia (PF&S) face a high risk of mobility disability. Early identification of predictors is essential to develop targeted preventive strategies. We performed a secondary analysis of the SPRINTT trial (NCT02582138) including community-dwelling older adults with PF&S and preserved mobility at baseline. To avoid intervention-related confounding, only participants in the control group were analyzed. Incident mobility disability was defined as inability to complete a 400-meter walk test within 15 minutes. Eighty-eight baseline variables (demographic, clinical, functional, social, and laboratory) were tested as candidate predictors using multivariate classification methods and machine learning (Partial Least-Squares Discriminant Analysis [PLS-DA], Random Forest, Gradient Boosting). Cox proportional hazards models were applied to confirm associations. Among 759 participants (71.3% women; median age 78 years), 354 (47%) developed mobility disability over 26 months. Model accuracy was similar across approaches (best PLS-DA: 67.6%). Baseline 400 m walk time was the strongest and most consistent predictor. In multivariate Cox regression, longer walking time, older age, lower SPPB score, and higher CES-D and SARC-F scores were independently associated with disability risk. Notably, depressive symptoms modified the impact of walking time (interaction p < 0.001): participants with both slow gait (≥480s) and CES-D ≥9 had the highest risk (HR = 2.69; 95% CI: 1.89–3.84). Kaplan–Meier curves confirmed the additive effect of gait speed and depression. Slower gait and depressive symptoms independently and synergistically predict mobility disability in older adults with PF&S. Simple cutoffs (400 m walk ≥480s, CES-D ≥9) may help identify high-risk individuals and inform preventive interventions.

